# Immunogenicity and safety of a proposed pegfilgrastim biosimilar MSB11455 versus the reference pegfilgrastim Neulasta^®^ in healthy subjects: A randomized, double‐blind trial

**DOI:** 10.1002/prp2.578

**Published:** 2020-04-25

**Authors:** Chris Wynne, Christian Schwabe, Emmanuelle Vincent, Armin Schueler, Janka Ryding, Martin Ullmann, Vishal Ghori, Radmila Kanceva, Michael Stahl

**Affiliations:** ^1^ Christchurch Clinical Studies Trust Ltd Christchurch New Zealand; ^2^ Auckland Clinical Studies Ltd Auckland New Zealand; ^3^ Fresenius Kabi SwissBioSim GmbH Eysins Switzerland; ^4^ Biostatistics Merck Healthcare Darmstadt Germany; ^5^ SVAR Life Science – Wieslab AB Lund Sweden

**Keywords:** antidrug antibodies, immunogenicity, MSB11455, pegfilgrastim, safety, tolerability

## Abstract

MSB11455 is a proposed biosimilar to the currently licensed reference pegfilgrastim (Neulasta^®^). This study was designed primarily to compare the immunogenicity of MSB11455 and Neulasta^®^. As secondary objectives, the safety and tolerability of MSB11455 and Neulasta^®^ were also compared. Healthy adult subjects were randomized to either MSB11455 or Neulasta^®^, stratified by antipolyethylene glycol (PEG) antibody status at screening and study site. Subjects received a single subcutaneous dose of MSB11455 or Neulasta^®^ (both 6 mg/0.6 mL) on day 1 of each of two study periods (same product in both periods), separated by a washout of 28‐35 days. Immunogenicity samples were taken predose and up to day 84 post–first dose. Noninferiority was confirmed if the upper limit of the exact one‐sided adjusted 95% confidence interval (CI) for the difference in antidrug antibody (ADA)‐positive rates was < 10%. Safety was assessed throughout the study. Overall, 336 subjects were randomized and treated (N = 168 in each group). Noninferiority of MSB11455 over Neulasta^®^ was demonstrated for immunogenicity; the difference in confirmed treatment‐induced ADA‐positive rate between MSB11455 and Neulasta^®^ was −0.6% (upper limit of the exact one‐sided adjusted 95% CI: 6.25%). ADAs were mostly directed against the PEG moiety of pegfilgrastim. No filgrastim‐specific neutralizing antibodies were detected in either treatment group. Safety and tolerability were as expected for pegfilgrastim, and comparable between treatments. This study supports and strengthens the available evidence for the biosimilarity of MSB11455 to Neulasta^®^.

**Abbreviations**
ADA: antidrug antibody
AE: adverse event
ANC: absolute neutrophil count
BMI: body mass index
CI: confidence interval
CTCAE: Common Terminology Criteria for Adverse Events
DTL: drug tolerance limit
ECG: electrocardiogram
EMA: European Medicines Agency
FDA: Food and Drug Administration
GCP: Good Clinical Practice
G‐CSF: granulocyte colony‐stimulating factor
ICH: International Council for Harmonisation
ITT: intent‐to‐treat
LPC: low positive control
MAb: monoclonal antibody
MCSF: macrophage colony‐stimulating factor
mPEG: methoxy‐polyethylene glycol
NAb: neutralizing antibody
PEG: polyethylene glycol
PP: per protocol
SAE: serious adverse event
SC: subcutaneous
SD: standard deviation
TEAE: treatment‐emergent adverse event
WBC: white blood cell

## 1 INTRODUCTION

Myelosuppressive chemotherapy is associated with the development of neutropenia, the consequences of which can be serious, with even minor infections potentially becoming life threatening. Clinically, chemotherapy‐induced neutropenia is defined as an absolute neutrophil count (ANC) of < 1.0x10^9^/L; at this ANC, the risk of infection begins to rise.^1^ Febrile neutropenia is defined as a temperature of > 38.2ºC on two determinations with severe neutropenia (ANC < 0.5 × 10^9^/L), and this event indicates a high likelihood of localized or systemic infection.^1^ Thus, persistent or severe neutropenia can be chemotherapy dose limiting, affecting the efficacy of these regimens. Treatment with a recombinant human granulocyte colony‐stimulating factor (G‐CSF), such as filgrastim, stimulates the proliferation, differentiation, and activation of neutrophils and reduces neutrophil maturation time.^2^

Pegfilgrastim is a pegylated form of filgrastim that requires administration only once per chemotherapy cycle.^3^ High‐level evidence indicates that prophylactic use of either filgrastim or pegfilgrastim improves the likelihood of completing dose‐dense and dose‐intense chemotherapy and allows a broader range of patients to be treated.^4^ Prophylactic use of G‐CSF in patients receiving myelosuppressive chemotherapy also reduces the risk of early mortality (ie, during the chemotherapy period), including that related to infection, in addition to reducing the risk of febrile neutropenia.^5^ The risk of febrile neutropenia, and its associated complications, is generally lower in patients receiving prophylaxis with pegfilgrastim than in those receiving filgrastim.^6^ Prophylactic use of these agents is, therefore, recommended in patients receiving a chemotherapy regimen with a high risk of febrile neutropenia and in situations where dose‐dense or dose‐intense chemotherapy strategies have survival benefits or when reductions in chemotherapy dose intensity or density are known to be associated with a poor prognosis.^4^, ^7^

Neulasta^®^ (pegfilgrastim; Amgen, Inc), the US reference product, is indicated to decrease the incidence of infection, as manifested by febrile neutropenia, in patients with nonmyeloid malignancies receiving myelosuppressive anticancer drugs associated with a clinically significant incidence of febrile neutropenia.^3^ MSB11455 is a proposed biosimilar to the currently licensed pegfilgrastim, Neulasta^®^. As with all therapeutic proteins, there is a risk of immunogenic reactions associated with administration of filgrastim or pegfilgrastim. The United States Food and Drug Administration (FDA) and European Medicines Agency (EMA) have both issued guidance on the development of biosimilars.^8^, ^9^ In this guidance, the clinical development program of a biosimilar must include a comparative clinical immunogenicity assessment. MSB11455 and Neulasta^®^ have analytical similarity of structural and functional attributes (data on file, Fresenius Kabi SwissBioSim GmbH, Switzerland), and have shown pharmacokinetic and pharmacodynamic equivalence in healthy volunteers.^10^ Therefore, this biosimilar and the reference product were expected to demonstrate a similar immunogenicity and safety profile.

This study, therefore, compared the immunogenicity of MSB11455 and Neulasta^®^ as the primary objective. As secondary objectives, the study also compared the safety and tolerability of the two pegfilgrastim products.

## 2 MATERIALS AND METHODS

### 2.1 Study design

This was a double‐blind, two‐dose, randomized, parallel‐group, group‐sequential, immunogenicity study (NCT03251339) in healthy subjects. The study was conducted at two sites in New Zealand during the period August 2017 to May 2018. Subjects were resident at a study site from day −1 to day 3 of each treatment period.

Subjects were randomized in a double‐blind manner to one of the two pegfilgrastim products, MSB11455 or Neulasta^®^ (Figure S1), stratified by antipolyethylene glycol (PEG) antibody status at screening (as preexisting PEG antibodies may be a confounding factor for antidrug antibodies [ADAs]). The study consisted of two treatment periods separated by a 28‐ to 35‐day washout period; during the first treatment period, subjects received either MSB11455 or Neulasta^®^, and during the second treatment period they received the same product. Subjects received a single subcutaneous injection of pegfilgrastim 6 mg/0.6 mL in the morning of day 1 of each of the two treatment periods, for a total of two injections. The end‐of‐treatment assessment visit occurred 28‐35 days after study drug administration in Period 2, whereas the end‐of‐study assessment visit was 84 ± 3 days after study drug administration in Period 1 or an early termination assessment visit.

The study was conducted in accordance with ethical principles of the International Council for Harmonisation (ICH) guideline for Good Clinical Practice (GCP) and the Declaration of Helsinki, as well as with applicable local regulations. All subjects provided written informed consent before study entry.

### 2.2 Study population

To be included in the study, participants (healthy men or healthy nonpregnant women) had to be aged ≥ 18 to ≤ 55 years with body mass index (BMI) ≥18.0 to ≤ 29.9 kg/m^2^. Eligible participants were in good health based on comprehensive medical history, physical examination, vital signs, and clinical laboratory tests, and had no known hypersensitivity to any component of either pegfilgrastim product. All subjects were required to comply with the contraception requirements specified in the clinical study protocol.

Potential participants were excluded based on usual exclusion criteria and the following: signs or symptoms of chronic obstructive pulmonary disease, smoking > 10 cigarettes/day, splenomegaly (spleen size > 13 cm in the craniocaudal dimension by ultrasound), prior exposure to any colony‐stimulating or growth factor in the 3 months before randomization, and prior exposure to therapeutic monoclonal antibodies (MAbs) targeting the bone marrow or blood cells; exposure to MAbs not affecting bone marrow or blood cells was allowed if the MAbs were discontinued > 3 months or five half‐lives (whichever was longer) before screening. Blood (≥500 mL) or plasma donation within 3 months, and stem cell or bone marrow donation within 12 months before screening were also not allowed.

### 2.3 Assessments

At screening, samples to determine anti‐PEG status were collected and evaluated using a kit‐based enzyme‐linked immunosorbent assay (ELISA). Samples for evaluating immunogenicity were taken predose on day 1 of Period 1, and postdose on day 13 of Period 1, day 28 of Period 1 (which also provided the predose sample for Period 2), days 13 and 28 of Period 2, and at the end‐of‐study assessment visit (84 ± 3 days after first drug administration in Period 1) or early termination assessment visit. Subjects who were positive for treatment‐induced ADAs and who did not have two subsequent ADA samples at baseline levels by the end‐of‐study assessment visit continued into immunogenicity follow‐up. During this follow‐up, samples were taken every 5 weeks ± 7 days until two consecutive ADA samples had returned to baseline.

All immunogenicity samples were evaluated in a bioanalytical laboratory using a validated ELISA for ADA status. In this ELISA, labeled MSB11455 was employed as capture and detection reagent. The ability of the assay to detect antibodies against MSB11455 and Neulasta^®^ was demonstrated during validation. All collected samples underwent a multitiered analytical approach to confirm the presence of ADA, collect semiquantitative information, and determine the binding specificity. First, a screening assay was used to detect potential ADA, followed by a confirmation assay, using MSB11455 as the competitive inhibitor, to reliably confirm the presence of ADA. Next, a titration assay was performed to collect semiquantitative information about the confirmed ADA, and finally a specificity assay was used to distinguish between ADAs binding to PEG and those binding to filgrastim. Relative sensitivity of the assay was determined using affinity‐purified polyclonal rabbit antipegfilgrastim and monoclonal mouse anti‐PEG antibodies. The determined relative sensitivities were 10 and 19 ng/mL, respectively. The determined drug tolerance limit (DTL) of the assay was 0.25 ng/mL MSB11455 at a low positive control (LPC) level of 15 ng/mL. Considering the half‐life of pegfilgrastim (15 to 80 hours), ^3^ the time between dosing and the first ADA sampling time point (13 days), and the results of a pharmacokinetic study (NCT03251248), the drug tolerance of the assay was considered adequate.

For ADA‐positive samples, NAb status was determined using a validated cell‐based assay that employed the cytokine‐dependent cell line M‐NFS‐60, a murine lymphoblastoid cell line derived from a myelogenous leukemia cell line and adapted to become filgrastim dependent (therefore, neutralization of filgrastim or pegfilgrastim will inhibit cell proliferation). However, macrophage colony‐stimulating factor (MCSF) neutralization also inhibits proliferation of this cell line. Thus, a tiered approach, as per regulatory guidance, was used to detect antibodies neutralizing pegfilgrastim and confirm their specificity toward filgrastim. In the first tier, ADA‐positive samples were assessed for neutralizing activity toward pegfilgrastim. In the second tier, neutralizing activity toward MCSF was tested to confirm the specificity of pegfilgrastim inhibition. Finally, samples neutralizing pegfilgrastim but not inhibiting MCSF‐induced cell proliferation were tested for their capacity to inhibit filgrastim. The determined DTL of this assay was 0.5 ng/mL MSB11455 and, based on the reasoning already presented for the ADA assay, the drug tolerance of the assay was considered adequate.

To avoid the confounding effect of pre‐existing antibodies, a subject was considered to have a confirmed treatment‐induced ADA‐positive status if one of two situations occurred. Firstly, if the predose sample was ADA negative and at least one postdose sample was positive in the ADA confirmatory assay. Secondly, if the predose sample was positive and at least one postdose sample was positive in the ADA confirmatory assay with a titer‐fold increase compared to predose above the minimum significant ratio (the minimum ratio between titers needed to declare a statistically significant difference), which was determined to be 3.6 during assay validation.

Safety and tolerability, including treatment‐emergent adverse events (TEAEs), injection site reactions, physical examination findings, vital signs, results of routine laboratory testing and 12‐lead electrocardiograms (ECGs), and concomitant medication data, were assessed from the time of signing informed consent and throughout the study. TEAEs of special interest were acute hypersensitivity (occurring within 48 hours after study drug administration); clinically significant increase in spleen size; ANC ≥ 75 × 10^9^/L (or white blood cell [WBC] count ≥ 90 × 10^9^/L); or signs and symptoms of hyperviscosity syndrome. Abdominal ultrasound was performed to assess spleen size before each dose and at the end‐of‐treatment assessment visit (or early termination assessment visit, as appropriate); further assessments of spleen size were allowed during the study if clinical signs or symptoms suggestive of splenic enlargement were noted.

### 2.4 Endpoints

The primary study endpoints were confirmed treatment‐induced ADA‐positive status to pegfilgrastim from predose on day 1 of Period 1 until the end‐of‐study assessment visit, and confirmed neutralizing antibody (NAb) status to pegfilgrastim from predose on day 1 of Period 1 until the end‐of‐study assessment visit. Secondary endpoints included safety and further immunogenicity endpoints.

Safety endpoints were occurrence of TEAEs, serious AEs (SAEs), AEs of special interest, and abnormal laboratory values and vital signs in subjects from the first administered dose until the end‐of‐study assessment visit. Additional immunogenicity endpoints were ADA status by time point, ADA titer over time, and NAb status by time point.

### 2.5 Statistical analyses

#### 2.5.1 Sample size and analysis population

A group‐sequential design with an unblinded interim analysis was implemented. A maximum of 404 subjects were to be randomized 1‐to‐1 to each treatment arm, unless the study was stopped at the interim analysis, which was planned after 336 subjects had been randomized. This sample size was calculated to have 90% power to declare MSB11455 no worse than Neulasta^®^, either at the interim or final analysis if the true confirmed ADA‐positive rate by the end‐of‐study assessment visit in the Neulasta^®^ arm was 12%, based on findings of a previous study (NCT02205320), and assuming a true difference in rates of 0%. Other key assumptions for these sample size calculations included a noninferiority margin of 10% and a type I error rate of ≤5% for a one‐sided test for noninferiority; this noninferiority margin corresponded to a maximum true confirmed ADA‐positive rate with MSB11455 of 22% under the assumption that the true confirmed ADA‐positive rate with Neulasta^®^ was 12% by the end‐of‐study assessment visit.

The unblinded interim analysis for futility (nonbinding) and noninferiority took place when exactly 336 subjects were randomized and had completed the end‐of‐study assessment visit/early termination visit. After review of the interim analysis data, an Independent Data Monitoring Committee recommended that the study be stopped for noninferiority, and the interim analysis became the final analysis.

The primary analysis of treatment‐induced ADA‐positive status up to end‐of‐study assessment visit/early termination visit was performed on the intent‐to‐treat (ITT) population, defined as all randomized patients. Other immunogenicity outcomes and safety were analyzed in all subjects who received at least one administration of study drug (MSB11455 or Neulasta^®^).

#### 2.5.2 Main analyses

Baseline subject demographics and characteristics were summarized descriptively. For the primary analysis of treatment‐induced ADA‐positive status up to end‐of‐study assessment visit/early termination visit, the difference in confirmed treatment‐induced ADA‐positive rates between MSB11455 and Neulasta^®^ was estimated along with the corresponding exact one‐sided adjusted 95% confidence interval [CI].^11^, ^12^ Given that the primary endpoint was binary and the associated rates could be less than 10%, the power and type I error rate calculations of the study design were assessed using exact calculations.

Secondary immunogenicity endpoints were reported as the proportion of subjects with confirmed treatment‐induced ADA‐positive status at each visit, with corresponding two‐sided 95% CI (using Clopper‐Pearson method); the denominator was the number of subjects with samples available at that visit. Summary statistics (median values) were used for ADA titers by visit. The proportion of subjects NAb positive to filgrastim for each visit was also calculated, the denominator being the number of subjects with samples analyzed (ie, subjects who were ADA positive). TEAEs were summarized by treatment and overall, as the number and proportion of subjects affected.

#### 2.5.3 Sensitivity analyses

Sensitivity analyses for the primary immunogenicity endpoint were conducted in the per protocol population (all subjects who received both doses of pegfilgrastim, had at least one ADA status available after day 1 of Period 2, and had no clinically important protocol deviations) and using a stratified analysis allowing for anti‐PEG antibody status at screening.

## 3 RESULTS

### 3.1 Patients

**Figure 1:**
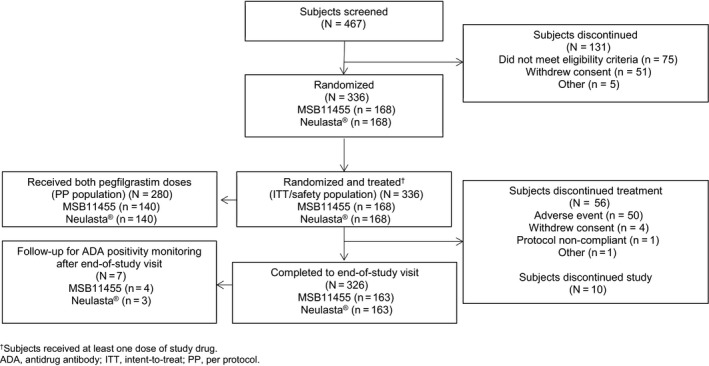
Subject disposition

A total of 336 subjects were randomized and treated (Figure 1). Baseline subject demographics and characteristics were well balanced between treatment arms (Table 1); overall, 4.8% of included subjects were anti‐PEG antibody positive at screening.

**Table 1:** Subject demographics and characteristics at baseline by treatment group and overall

	MSB11455 (N = 168)	Neulasta^®^ (N = 168)	Overall (N = 336)
Male, n (%)	100 (59.5)	91 (54.2)	191 (56.8)
Female, n (%)	68 (40.5)	77 (45.8)	145 (43.2)
Race, n (%)			
White	120 (71.4)	131 (78.0)	251 (74.7)
Black/African American	3 (1.8)	2 (1.2)	5 (1.5)
Asian	16 (9.5)	13 (7.7)	29 (8.6)
Native Hawaiian or other Pacific Islander	5 (3.0)	2 (1.2)	7 (2.1)
American Indian or Alaska Native	1 (0.6)	0	1 (0.3)
Other	23 (13.7)	20 (11.9)	43 (12.8)
Ethnicity, n (%)			
Hispanic	9 (5.4)	15 (8.9)	24 (7.1)
Japanese	1 (0.6)	2 (1.2)	3 (0.9)
Anti‐PEG antibody positive^a^, n (%)	8 (4.8)	8 (4.8)	16 (4.8)
Age (years), mean ± SD	26 ± 7.1	28 ± 7.9	27 ± 7.5
Weight (kg), mean ± SD	72.6 ± 11.4	72.3 ± 10.8	72.4 ± 11.1
BMI (kg/m^2^), mean ± SD	24.2 ± 2.7	24.3 ± 2.8	24.2 ± 2.8

Abbreviations: BMI, body mass index; PEG, polyethylene glycol; SD, standard deviation.

^a^ At screening.

### 3.2 Immunogenicity

**Figure 2:**
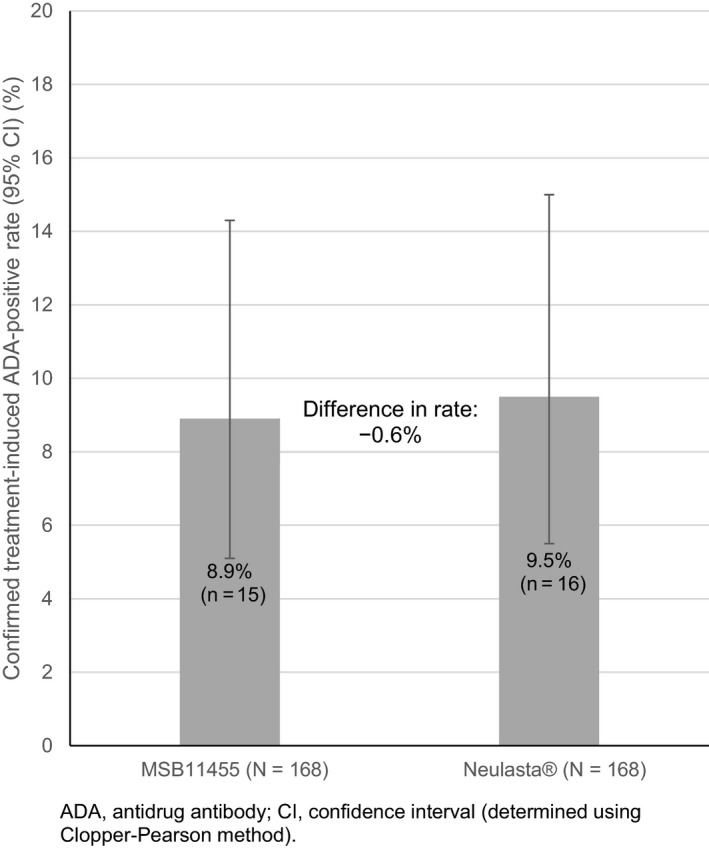
Proportion of healthy subjects with treatment‐induced ADA‐positive status at any time after the first of two single doses of MSB11455 or Neulasta^®^ administered 28‐35 days apart until the end‐of‐study assessment visit/early termination visit

Noninferiority of MSB11455 over Neulasta^®^ was demonstrated for immunogenicity (upper limit of the exact one‐sided adjusted 95% CI < 10%). The confirmed treatment‐induced ADA‐positive rate was similar with both pegfilgrastim products (Figure 2), with a difference in rate between MSB11455 and Neulasta^®^ of −0.6% (upper limit of the exact one‐sided adjusted 95% CI: 6.25). The overall postdose ADA‐positive rate, not constrained to a treatment‐induced ADA‐positive status, was also comparable between treatments. Over the entire study period, 15 subjects (8.9%) who received MSB11455 and 18 (10.7%) who received Neulasta^®^ had an ADA‐positive status at any time.

**Figure 3:**
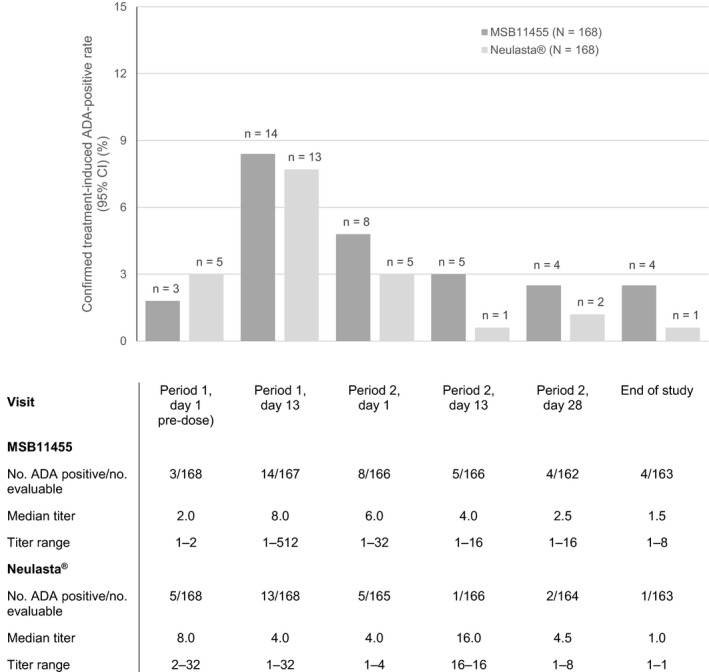
Summary of confirmed ADA‐positive status results over time from before the first of two single doses of MSB11455 or Neulasta^®^ administered 28‐35 days apart until the end‐of‐study assessment visit/early termination visit in healthy subjects: proportion of ADA‐positive subjects,^†^ with ADA titers in ADA‐positive subjects^‡^ presented below the figure

Treatment‐induced ADA‐positive rates over time were also comparable between treatments (Figure 3). The highest positivity rate was observed on day 13 of Period 1, and rates decreased thereafter. Although there were numerically more ADA‐positive subjects in the MSB11455 group than in the Neulasta^®^ group in period 2, the overall positivity rate in period 2 was low (<5.0% in each group). Overall, there were no relevant differences in median ADA titers over time between the two treatment groups among subjects with an ADA‐positive sample (Figure 3). At most visits, the ratio of median titers in each treatment group was below the minimum significant ratio (3.6), and hence not significantly different. On day 13 of Period 2, the difference for MSB1145 vs Neulasta^®^ was above the minimum significant ratio, but that a single subject was ADA positive in the Neulasta^®^ group prevents any conclusions regarding the clinical relevance of this finding. ADAs were mostly directed against the PEG moiety of pegfilgrastim, with no relevant differences in specificity observed between the two treatment groups (Table 2). No filgrastim‐specific NAbs were detected in either treatment group, but four subjects (three after MSB11455 and one after Neulasta^®^) had at least one postdose sample with detectable pegfilgrastim neutralizing activity.

**Table 2:** Specificity of ADAs in subjects who were ADA positive at any time after the first of two single doses of MSB11455 or Neulasta^®^ administered 28‐35 days apart until the end‐of‐study assessment visit/early termination visit in healthy subjects

n (%)	MSB11455 (N = 168)	Neulasta^®^ (N = 168)	Overall (N = 336)
mPEG‐positive	10 (6.0)	12 (7.1)	22 (6.5)
Filgrastim‐positive	0	1 (0.6)	1 (0.3)
mPEG‐ and filgrastim‐positive^a^	4 (2.4)	1 (0.6)	5 (1.5)
mPEG‐ and filgrastim‐negative^a^	1 (0.6)	4 (2.4)	5 (1.5)

Abbreviations: ADA, antidrug antibody; mPEG, methoxy‐polyethylene glycol.

^a^ All ADAs associated with a low titer

Seven subjects identified as treatment‐induced ADA positive did not have two consecutive ADA samples that had returned to baseline by the end‐of‐study assessment visit, and therefore underwent immunogenicity follow‐up, consisting of a scheduled visit every 5 weeks (four subjects in the MSB11455 group and three in the Neulasta^®^ group). All six evaluable subjects (one subject treated with Neulasta^®^ was lost to follow‐up) had two consecutive ADA samples that returned to baseline (ie, subject completed follow‐up) within a maximum of five visits. In the MSB11455 group, one subject required a maximum of five follow‐up visits, two required three visits, and one required two visits. In the Neulasta^®^ group, the two evaluable subjects each required one follow‐up visit.

### 3.3 Sensitivity analyses

Sensitivity analyses conducted either in the per protocol population or with stratification for anti‐PEG antibody status at screening confirmed the primary analysis results, showing the noninferiority of MSB11455 over Neulasta^®^ for immunogenicity (data not shown).

### 3.4 Safety and tolerability

Safety and tolerability were similar between treatments (Table 3). There were no deaths and the numbers of reported SAEs were small with no notable differences between treatments. TEAEs leading to discontinuation of study drug occurred with similar frequency in both treatment groups (Table 3) and were most commonly an increased WBC count to ≥ 50.0 × 10^9^/L (9.5% of MSB11455 and 11.9% of Neulasta^®^ recipients), which was initially a protocol prespecified treatment withdrawal criterion. TEAEs were generally mild to moderately severe, self‐limiting, and resolved without sequelae. No TEAEs were of National Cancer Institute Common Terminology Criteria for Adverse Events (CTCAE) grade ≥ 4. The most common TEAEs were musculoskeletal and connective tissue disorders and nervous system disorders (Table 3).

**Table 3:** Safety and tolerability of two single doses of MSB11455 or Neulasta^®^ administered 28‐35 days apart in healthy subjects

	MSB11455 (N = 168)	Neulasta^®^ (N = 168)
Any TEAE	167 (99.4)	167 (99.4)
Any study drug‐related TEAE	166 (98.8)	158 (94.0)
Any serious TEAE	1 (0.6)	2 (1.2)
Any study drug‐related serious TEAE	1 (0.6)^a^	1 (0.6)^b^
Any Grade ≥ 3^c^ TEAE	1 (0.6)	2 (1.2)
Any study drug‐related Grade ≥ 3^c^ TEAE	0	0
Death	0	0
Any TEAE leading to discontinuation of study drug	25 (14.9)	25 (14.9)
AE of special interest	25 (14.9)	30 (17.9)
Splenomegaly	2 (1.2)	0
Drug hypersensitivity	0	2 (1.2)
White blood cells increased	23 (13.7)	27 (16.1)
Drug eruption	0	1 (0.6)
Most common treatment‐emergent AEs (>5% of subjects)		
Headache	105 (62.5)	120 (71.4)
Bone pain	113 (67.3)	101 (60.1)
Spinal pain	67 (39.9)	68 (40.5)
Upper respiratory tract infection	32 (19.0)	20 (11.9)
Nausea	32 (19.0)	19 (11.3)
White blood cell count increased^d^	23 (13.7)	27 (16.1)
Myalgia	19 (11.3)	17 (10.1)
Vomiting	18 (10.7)	9 (5.4)
Musculoskeletal chest pain	12 (7.1)	17 (10.1)
Abdominal pain	9 (5.4)	15 (8.9)
Diarrhea	8 (4.8)	15 (8.9)
Oropharyngeal pain	12 (7.1)	14 (8.3)
Injection site bruising	12 (7.1)	10 (6.0)
Arthralgia	11 (6.5)	11 (6.5)
Dizziness	11 (6.5)	11 (6.5)
Contusion	11 (6.5)	7 (4.2)
Fatigue	7 (4.2)	11 (6.5)
Back pain	8 (4.8)	9 (5.4)

Note

Data shown are n (%) of subjects.

Abbreviations: AE, adverse event; TEAE, treatment‐emergent adverse event.

^a^ Acute febrile neutrophilic dermatosis

^b^ Spontaneous abortion in partner

^c^ National Cancer Institute—Common Terminology Criteria for Adverse Events grade, Version 4.03

^d^ All events considered related to study drug

AEs of special interest were reported in similar proportions of subjects in the MSB11455 and Neulasta^®^ groups (Table 3). Both instances of an increase in spleen size (coded as splenomegaly) (Grade 1 and 2, respectively) – only one of which was considered by the investigator as clinically significant on splenic ultrasound examination – resolved spontaneously and the subjects completed the study. Injection site reactions occurred in similar proportions of MSB11455 and Neulasta^®^ recipients (11.9% vs 9.5%), the most common event being bruising.

Changes in laboratory values and vital signs were similar between the two treatments. Eight subjects in each treatment group were anti‐PEG positive at screening; the frequency of TEAEs reported after administration of each pegfilgrastim product was comparable in these subjects. Similarly, there were no apparent differences in the pattern of TEAEs reported for ADA‐positive subjects among MSB11455 and Neulasta^®^ recipients.

## 4 DISCUSSION

As therapeutic proteins, both MSB11455 and Neulasta^®^ were expected to induce ADAs.^3^ Although, existing data suggest a low immunogenic potential for pegfilgrastim,^3^ the ability to stimulate antibody formation can differ between structurally closely related molecules, with even minor changes affecting immunogenicity.^13^, ^14^ ADAs have the potential to adversely interfere with the therapeutic action of, or reduce or prolong exposure to, the therapeutic agent and/or predispose to adverse reactions.^13^, ^14^ In the case of filgrastim/pegfilgrastim, ADAs with filgrastim neutralizing capacity are particularly critical due to potential cross‐reactivity with the endogenous counterpart. Demonstration of immunogenic similarity of a biosimilar to the reference product in a head‐to‐head comparison is, therefore, a critical parameter for defining the safety of the biosimilar.^13^

The design of this study was appropriate for comparing the immunogenicity and safety profiles of MSB11455 and Neulasta^®^, with both the study design and population in line with regulatory guidance. The parallel‐group design of the study ensured that immunogenicity assessments were related to only one treatment. This study was conducted in healthy subjects because they are the most homogenous population for a sensitive assessment of immunogenicity; their immune system is not affected by chemotherapy (thereby improving sensitivity to detect differences in immunogenicity), and animal models have low predictive value with respect to immunogenicity in humans.^13^ The dose of study drug administered is the approved dose for Neulasta^®^ when used in sequence with cytotoxic chemotherapy.^3^ The 28‐day period between doses was appropriate as pegfilgrastim is administered once per chemotherapy cycle, and given the half‐life of pegfilgrastim, the time between dosing and the first ADA sampling time point was considered adequate. Two injections were considered sufficient to assess immunogenicity based on unpublished historical data (from study NCT02205320), where the majority (approximately 95%) of immunogenicity occurred before a third dose of pegfilgrastim was given (ie, within 10 weeks of the first dose) and was mostly transient. Sampling time points were designed to identify potential early (Day 13) and late (Day 28) immune responses. In this study, ADA‐positive rates peaked within 2 weeks after the first dose of both pegfilgrastim products, with a later incidence and titer decline, suggesting that a longer study period would not be more informative.

In this study, noninferiority of MSB11455 over Neulasta^®^ was demonstrated for the rate of confirmed treatment‐induced ADA‐positive status: the treatment‐induced confirmed immunogenicity rate was 8.9% for MSB11455% vs 9.5% for Neulasta^®^ (difference in rate: −0.6%; upper limit of the exact one‐sided adjusted 95% CI: 6.25). Furthermore, no relevant differences in ADA characteristics such as onset, titer, and specificity were found between the treatment groups. Although there was a numerical difference in the number of ADA‐positive subjects in the MSB11455 group compared with the Neulasta^®^ group in period 2, the small difference between the treatment arms in ADA incidence at individual visits (1.2% to 2.4%) was not conclusive and did not affect the demonstrated noninferiority in terms of immunogenicity of MSB11455 compared with Neulasta^®^ at the overall study level. Most detected ADAs with both treatments were directed against the PEG moiety of pegfilgrastim. No filgrastim‐specific NAb was detected in either treatment arm, in line with the observed ADA specificity findings. Findings of the main analyses were supported by those of sensitivity analyses, including those performed in the per protocol population that included all subjects who received both doses of pegfilgrastim.

Study results also showed that the safety and tolerability of MSB11455 and Neulasta^®^ were similar, and were as expected for administration of pegfilgrastim.^3^ Bone pain and headache were among the most frequently reported TEAEs for both treatments, which is expected with pegfilgrastim treatment.^3^ Pegfilgrastim is also associated with musculoskeletal pain because of bone marrow remodeling and increased precursor turnover.^15^ Splenic rupture has been reported following administration of filgrastim^16^ or pegfilgrastim^3^; therefore, the spleen was monitored throughout the study. Two subjects experienced a mild‐to‐moderate increase in spleen size; of these only one was deemed by the investigator as clinically significant on splenic ultrasound examination. In both instances, this spontaneously resolved. A relatively large number of subjects reported an increase in WBC count to ≥ 50.0 x 10^9^/L as an AE of special interest, which resulted in treatment discontinuations in these subjects (as per corresponding withdrawal criterion prespecified in the initial version of the clinical study protocol). However, all these events were self‐limiting, returning spontaneously to normal levels within 10 days. The initial WBC count ‘safety threshold’ defined in the initial version of the clinical study protocol was at a level too low for healthy volunteers who had intact bone marrow. The study protocol was, therefore, amended to reflect the pronounced increment in WBC count expected in healthy subjects following stimulation by pegfilgrastim (to a WBC count ≥ 90.0 x 10^9^/L) and no further events of this nature were reported after this amendment.

## 5 CONCLUSION

MSB11455 and Neulasta^®^ showed comparable immunogenicity, safety, and tolerability in healthy subjects, and MSB11455 was noninferior to Neulasta^®^ for immunogenicity, in terms of treatment‐induced ADA‐positive rates. This study supports and strengthens available evidence for the biosimilarity of MSB11455 to Neulasta^®^.

## DISCLOSURES

Chris Wynne is a shareholder in Christchurch Clinical Studies Trust Ltd; Christian Schwabe is an employee and shareholder in ACS; Emmanuelle Vincent, Michael Stahl, Martin Ullmann, Radmila Kanceva, and Vishal Ghori are employees of Fresenius Kabi SwissBioSim GmbH, Switzerland; Janka Ryding was employed by Fresenius Kabi SwissBioSim GmbH at the time of study conduct and is currently an employee of SVAR Life Science AB, Sweden; Armin Schueler is an employee of Merck Healthcare, Darmstadt, Germany.

## AUTHOR CONTRIBUTIONS

Chris Wynne: Study design, acquisition and interpretation of data, critical revision of the manuscript for important intellectual content, final approval, and has participated sufficiently in the work to agree to be accountable for all aspects of the work in ensuring that questions related to the accuracy or integrity of any part of the work are appropriately investigated and resolved. Christian Schwabe: Acquisition of data, critical revision of the manuscript for important intellectual content, final approval, and has participated sufficiently in the work to agree to be accountable for all aspects of the work in ensuring that questions related to the accuracy or integrity of any part of the work are appropriately investigated and resolved. Emmanuelle Vincent: Conception and study design, acquisition, analysis and interpretation of data, critical revision of the manuscript for important intellectual content, final approval, and has participated sufficiently in the work to agree to be accountable for all aspects of the work in ensuring that questions related to the accuracy or integrity of any part of the work are appropriately investigated and resolved. Armin Schueler: Study design, analysis and interpretation of data, critical revision of the manuscript for important intellectual content, final approval, and has participated sufficiently in the work to agree to be accountable for all aspects of the work in ensuring that questions related to the accuracy or integrity of any part of the work are appropriately investigated and resolved. Janka Ryding: Conception and study design, acquisition and analysis of data, critical revision of the manuscript for important intellectual content, final approval, and has participated sufficiently in the work to agree to be accountable for all aspects of the work in ensuring that questions related to the accuracy or integrity of any part of the work are appropriately investigated and resolved. Martin Ullmann: Study design, analysis and interpretation of data, critical revision of the manuscript for important intellectual content, final approval, and has participated sufficiently in the work to agree to be accountable for all aspects of the work in ensuring that questions related to the accuracy or integrity of any part of the work are appropriately investigated and resolved. Vishal Ghori: Analysis and interpretation of data, critical revision of the manuscript for important intellectual content, final approval, and has participated sufficiently in the work to agree to be accountable for all aspects of the work in ensuring that questions related to the accuracy or integrity of any part of the work are appropriately investigated and resolved. Radmila Kanceva: Acquisition, analysis and interpretation of data, drafting and critical revision of the manuscript for important intellectual content, final approval, and has participated sufficiently in the work to agree to be accountable for all aspects of the work in ensuring that questions related to the accuracy or integrity of any part of the work are appropriately investigated and resolved. Michael Stahl: Conception of work, acquisition, analysis, and interpretation of data, critical revision of the manuscript for important intellectual content, final approval, and has participated sufficiently in the work to agree to be accountable for all aspects of the work in ensuring that questions related to the accuracy or integrity of any part of the work are appropriately investigated and resolved.

## Supporting information

Fig S1
