# Supplementary figures and images for: Immunogenicity and safety of a proposed pegfilgrastim biosimilar MSB11455 versus the reference pegfilgrastim Neulasta® in healthy subjects: A randomized, double‐blind trial
Source: Pharmacol Res Perspect. 2020 Apr 25;8(2):e00578. doi: 10.1002/prp2.578 (PMC7183238; doi:10.1002/prp2.578)

Supplementary Figure 1      Study design

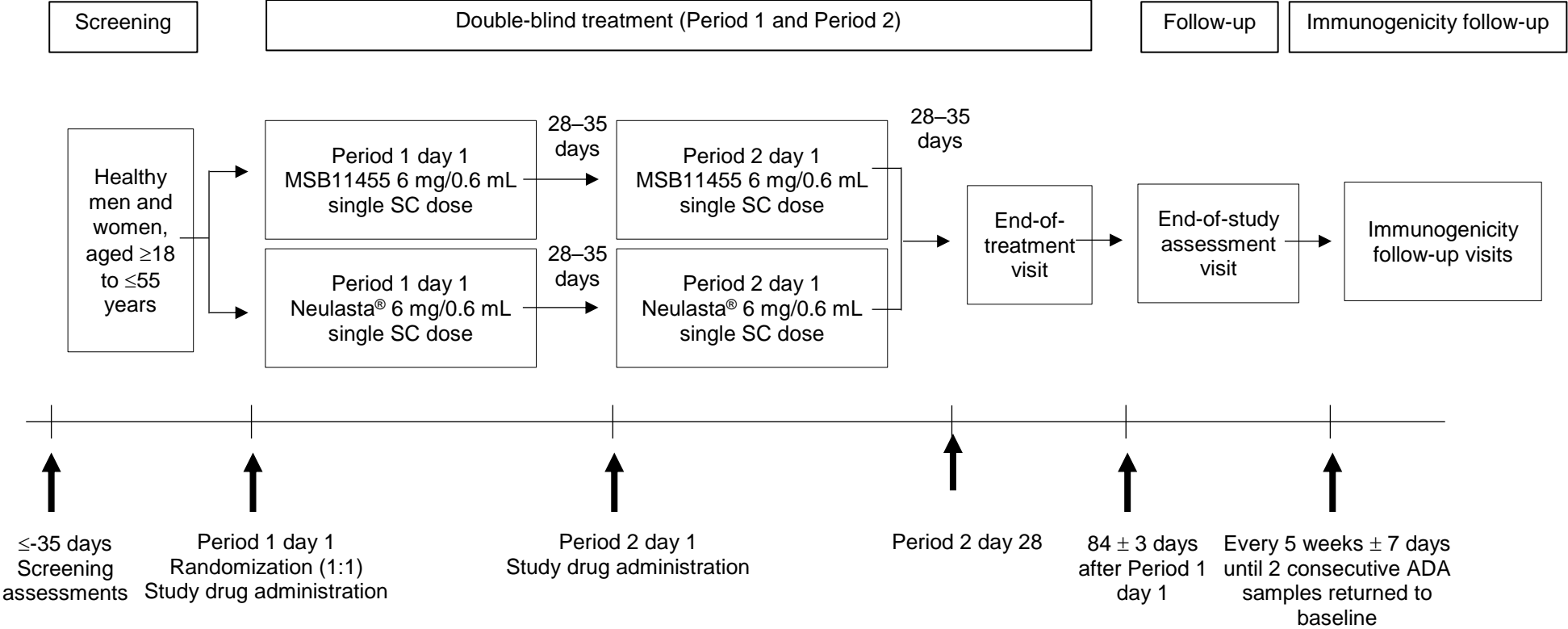

ADA, antidrug antibodies; SC, subcutaneous

Supplement: Supplementary file 1 — Fig S1 [file PRP2-8-e00578-s001.pdf]
